# Blockade of TRPV1 Inhibits Methamphetamine-induced Rewarding Effects

**DOI:** 10.1038/s41598-018-19207-2

**Published:** 2018-01-17

**Authors:** Yu-Hua Tian, Shi-Xun Ma, Kwang-Wook Lee, Sunmee Wee, George F. Koob, Seok-Yong Lee, Choon-Gon Jang

**Affiliations:** 1 Department of Pharmacology, School of Pharmacy, Qingdao University, Qingdao, 266021 China; 2 Department of Pharmacology, School of Pharmacy, Sungkyunkwan University, Suwon, 16419 Republic of Korea; 3 Committee on the Neurobiology of Addictive Disorders, The Scripps Research Institute, La Jolla, CA 92037 USA; 4 Neurobiology of Addiction Section, National Institute on Drug Abuse, National Institutes of Health, Baltimore, MD 21224 USA

## Abstract

Methamphetamine (MAP) is the most widely used psychostimulant in the world, but the exact mechanisms underlying MAP addiction are not yet fully understood. Recent studies have identified the distribution of TRPV1 in several brain regions that are related to drug addiction, including nucleus accumbens (NAc) and dorsal striatum (DSt). In the present study, we performed conditioned place preference (CPP) and self-administration tests to examine the effects of capsazepine (CPZ) and SB366791 (SB) on MAP reward. We found that both CPZ and SB significantly inhibited MAP-induced CPP and self-administration; in contrast, TRPV1 knock-out (KO) mice did not develop MAP-induced CPP. Real-time RT-PCR, Western blot and quantitative autoradiographic tests showed up-regulation of TRPV1 mRNA and protein expression in the NAc and/or DSt regions of mice exhibiting MAP-induced CPP. In addition, an *in vivo* microdialysis experiment showed that CPZ dramatically reduced dopamine (DA) levels in the NAc region of MAP-treated mice. Furthermore, attenuated dopamine transporter (DAT) binding levels in the NAc and DSt regions of MAP-induced CPP mice were reversed by CPZ. Together, these data suggest that TRPV1 plays an important role in MAP reward via the modulation of DA release and DAT density, thereby providing a novel therapeutic target for MAP addiction.

## Introduction

The transient receptor potential vanilloid subtype 1 channel (TRPV1) was first identified in peripheral afferent fibers as the receptor for capsaicin, the pungent substance present in chili peppers. TRPV1 is a nonselective cation channel activated by endogenous lipids, noxious heat, low pH, and endovanilloids, along with capsaicin^1,2^. Although TRPV1 expression in the brain is well documented^3^, its roles in health and disease are just beginning to be explored. Growing evidence indicates that TRPV1 may be associated with the neuronal and behavioral adaptations induced by addictive drugs, including anxiety, depression, drug consumption, and drug-seeking behaviors. Blocking TRPV1 is known to increase anxiety-like behaviors in rats^4,5^, and TRPV1 agonists have shown antidepressant-like effects in nicotine-induced depression-like behaviors^6^. In two previous studies, TRPV1 KO mice displayed reduced anxiety and conditioned fear^7^ and an alteration in the effects of ethanol on behavior^8^. Blockade of TRPV1 can attenuate morphine tolerance^9,10^ and inhibit morphine conditioned place preference (CPP) in rodents^11,12^. In addition, blocking TRPV1 also suppresses cocaine-seeking behavior in a cocaine-primed relapse^13^. Moreover, our previous work found that repeated methamphetamine (MAP) treatments can induce up-regulation of TRPV1 mRNA expression in the frontal cortex^14^. Taken together, these data indicate that TRPV1 could be a target of the rewarding effects of MAP.

MAP is a psychostimulant and one of the most problematic addictive drugs in the world^15^. The psychostimulatory effects of MAP are related to an increase in extracellular dopamine (DA) level in the brain by facilitating the release of DA and inhibiting DA reuptake^16^. Nonetheless, a growing number of studies have identified the importance of other neurotransmitters and related receptors, including glutamate, GABA, 5-HT, acetylcholine, and endocannabinoids, in the reward effect of MAP. Therefore, the exact mechanisms underlying MAP addiction are not yet fully understood. One study reported that anandamide, an endogenous cannabinoid receptor ligand, was involved in MAP drug-seeking behavior^17^. Interestingly, anandamide is also a ligand for TRPV1 receptors and activates this channel with a potency and efficacy that are lower than those exhibited at CB1 receptors^2^. Both findings indicate the possible engagement of TRPV1 in MAP abuse.

Therefore, in the present study, we performed CPP and self-administration tests to determine whether TRPV1 expression in the brain was altered by repeated MAP treatments and to assess the role of TRPV1 in MAP reward in mice. Furthermore, we studied possible mechanisms to explain how TRPV1 may contribute to MAP reward.

## Results

### Effects of CPZ and SB on the development of MAP-induced CPP

**Figure 1:**
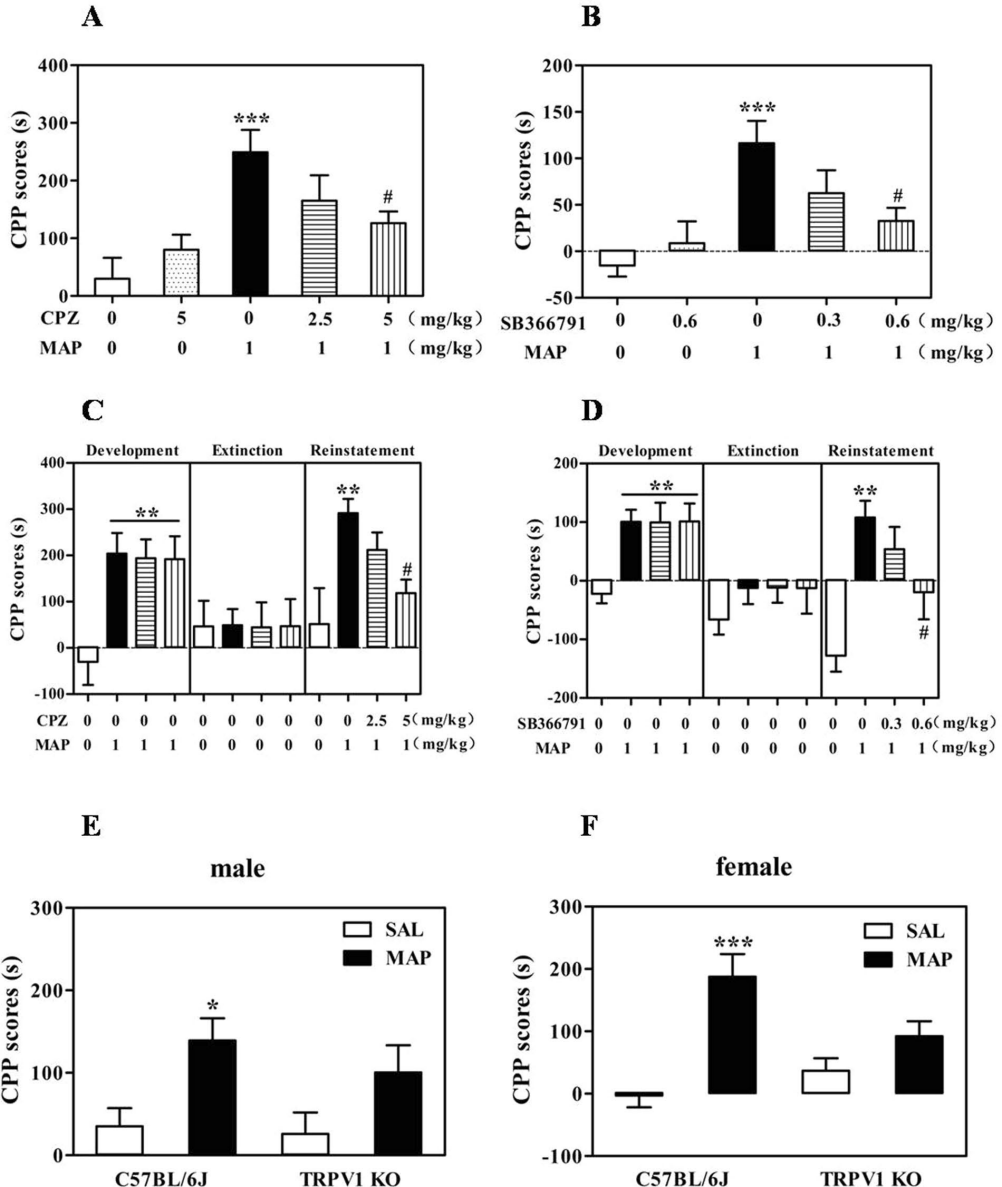
Role of TRPV1 in MAP-induced CPP. Inhibitory effects of CPZ and SB on the development (**A**,**B**) and drug-primed reinstatement (**C**,**D**) of MAP-induced CPP (development: ^***^P < 0.001 vs. saline control group, ^#^*P* < 0.01 vs. MAP control group; drug-primed reinstatement: ^**^P < 0.01 vs. saline control group, ^#^*P* < 0.05 vs. MAP control group). Deletion of TRPV1 partially prevented MAP-induced CPP development in both males (**E**) and females (**F**) (^**^P < 0.01, ^***^P < 0.001 vs. wild-type saline control group). Results are presented as the mean ± SEM (n = 10–13). Statistical analysis was performed by one-way ANOVA with Newman-Keuls *post hoc* test. SAL, saline; MAP, methamphetamine; CPZ, capsazepine.

The groups treated with CPZ 5 mg/kg or SB 0.6 mg/kg (i.p.) alone did not show any sign of CPP compared with the vehicle group. However, CPZ or SB administered 30 min before MAP injection decreased MAP-induced CPP [F_(4,47)_ = 6.18, *P* < 0.001; F_(4,51)_ = 6.09, *P* < 0.001]. This inhibitory effect appeared to be dose-dependent. Specifically, the group pretreated with either CPZ (5 mg/kg) or SB (0.6 mg/kg) showed a marked inhibition of MAP-induced CPP (*P* < 0.05). At a lower dose, CPZ (2.5 mg/kg) and SB (0.3 mg/kg) still inhibited MAP-induced CPP compared with the MAP group, but the effects were not statistically significant (Fig. 1A,B).

### Effects of CPZ and SB on the drug-primed reinstatement of MAP-induced CPP

As shown in Fig. 1C and D, one-way ANOVA revealed that the three groups treated with MAP produced a significant CPP compared with the saline control group [CPZ: F_(3,46)_ = 5.858, *P* < 0.05; SB: F_(3,44)_ = 5.444, *P* < 0.05]. However, this CPP was eliminated by a 7-day extinction phase, suggesting that the animals failed to show a preference for the MAP-paired compartment [CPZ: F_(3,46)_ = 0.7335, *P* > 0.05; SB: F_(3,44)_ = 0.4399, *P* > 0.05]. After the extinction of CPP was identified, on the next day, we carried out the MAP-primed reinstatement test with mice that remained extinguished to CPP after 7–8 days of saline injection. Two doses of CPZ (2.5 and 5 mg/kg, i.p.) or SB (0.3 and 0.6 mg/kg, i.p.) were administered 30 min before MAP injection. Only CPZ 5 mg/kg or SB 0.6 mg/kg showed a significant inhibitory effect on MAP-induced CPP reinstatement (*P* < 0.05, Newman-Keuls test) compared with the MAP groups; CPZ 2.5 mg/kg and SB 0.3 mg/kg did not show any significant inhibition. Based on these observations, we concluded that both CPZ and SB suppressed development and drug-primed reinstatement of MAP-induced CPP in a similar manner.

### Development of MAP-induced CPP in TRPV1 wild-type and KO mice

To examine the role of TRPV1 in the development of MAP-induced CPP, we conducted CPP tests with C57BL/6 J wild-type and TRPV1 KO mice, including both males and females. Mice were divided into two groups, with one group receiving saline and the other group receiving MAP. As shown in Fig. 1E, male TRPV1 KO mice treated with MAP (1 mg/kg, i.p.) did not produce CPP compared with the saline control group, whereas C57BL/6J wild type mice treated with MAP induced significant CPP compared with the saline control group (*P* < 0.05, Newman-Keuls test), indicating an important function of TRPV1 on the development phase of MAP-induced CPP [F_(3,43)_ = 3.932, *P* < 0.05]. Similarly, in females, C57BL/6J wild-type mice treated with MAP showed significant CPP compared with the saline control group (*P* < 0.001, Newman-Keuls test), but TRPV1 KO mice treated with MAP did not [F_(3,43)_ = 10.28, *P* < 0.01; Fig. 1F]. Therefore, there is no apparent gender dependence in TRPV1 involvement of MAP-induced CPP.

### Effects of SB on acquisition of MAP self-administration

**Figure 2:**
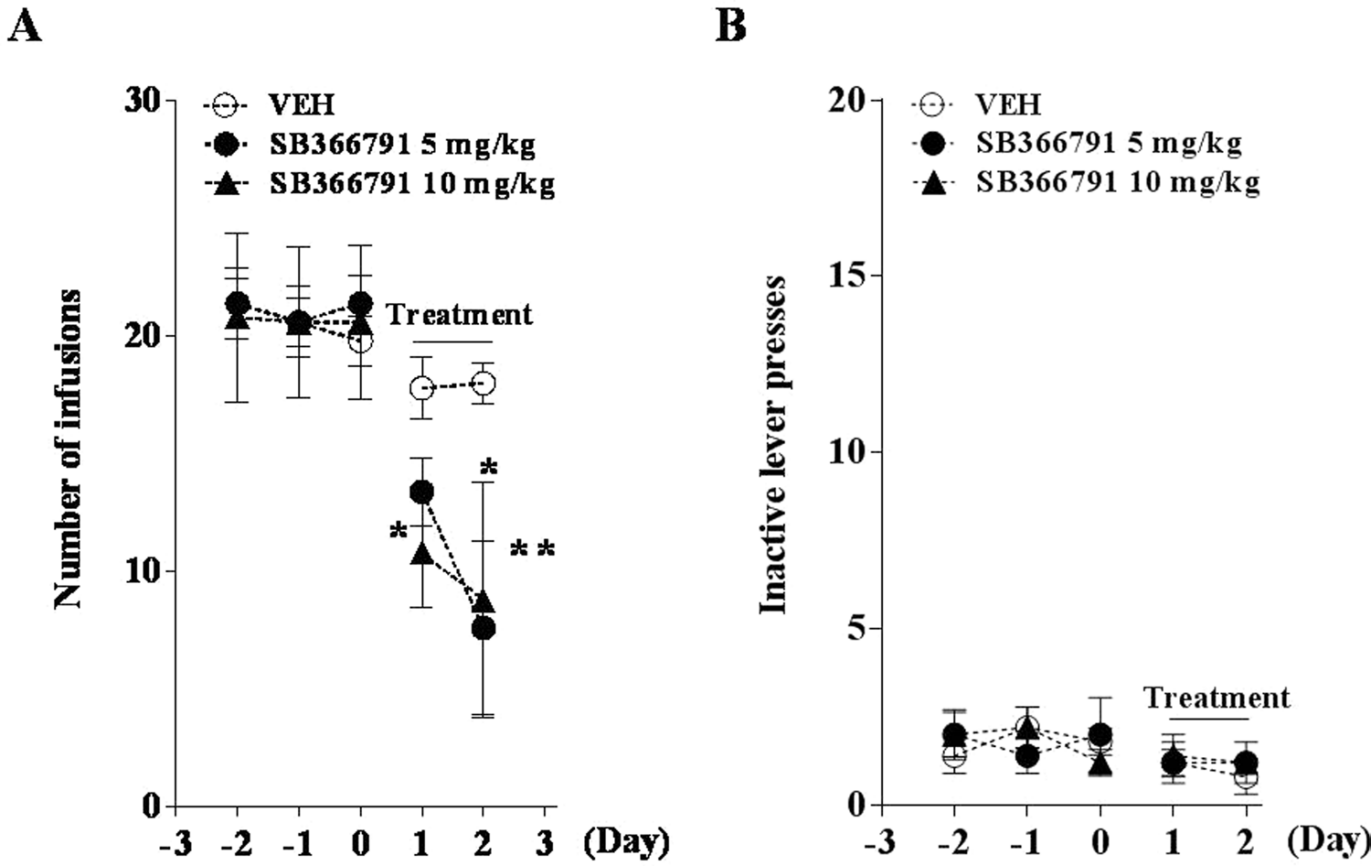
Effects of SB on acquisition of MAP self-administration on a fixed-ratio 1 (FR 1) schedule of reinforcement during a 2-h session. Following training sessions, rats were administered either vehicle or SB at 5 or 10 mg/kg intraperitoneally 30 min before the test sessions. Results show the mean number of infusions (**A**) or inactive lever pressing (**B**) during MAP training and test sessions (n = 5). ^*^P < 0.05, ^**^P < 0.01 compared with the vehicle group of each test session.

We first determined whether SB could decrease MAP-reinforced lever pressing. Systemic injection of 5 or 10 mg/kg SB significantly decreased MAP self-administration on a fixed ratio 1 (FR1) schedule of reinforcement. The number of infusions was significantly decreased by SB in the first and second test session, and it was decreased in a dose-dependent manner in the first session (Day 1) [F_(8,48)_ = 2.916, *P* < 0.01; Fig. 2A]. However, there was no difference in inactive lever pressing between these two groups [F_(8,48)_ = 0.3981, *P* > 0.05; Fig. 2B]. These data suggested that TRPV1 antagonism was involved in the acquisition process of MAP self-administration.

### Effects of CPZ and SB on relapse to MAP seeking

**Figure 3:**
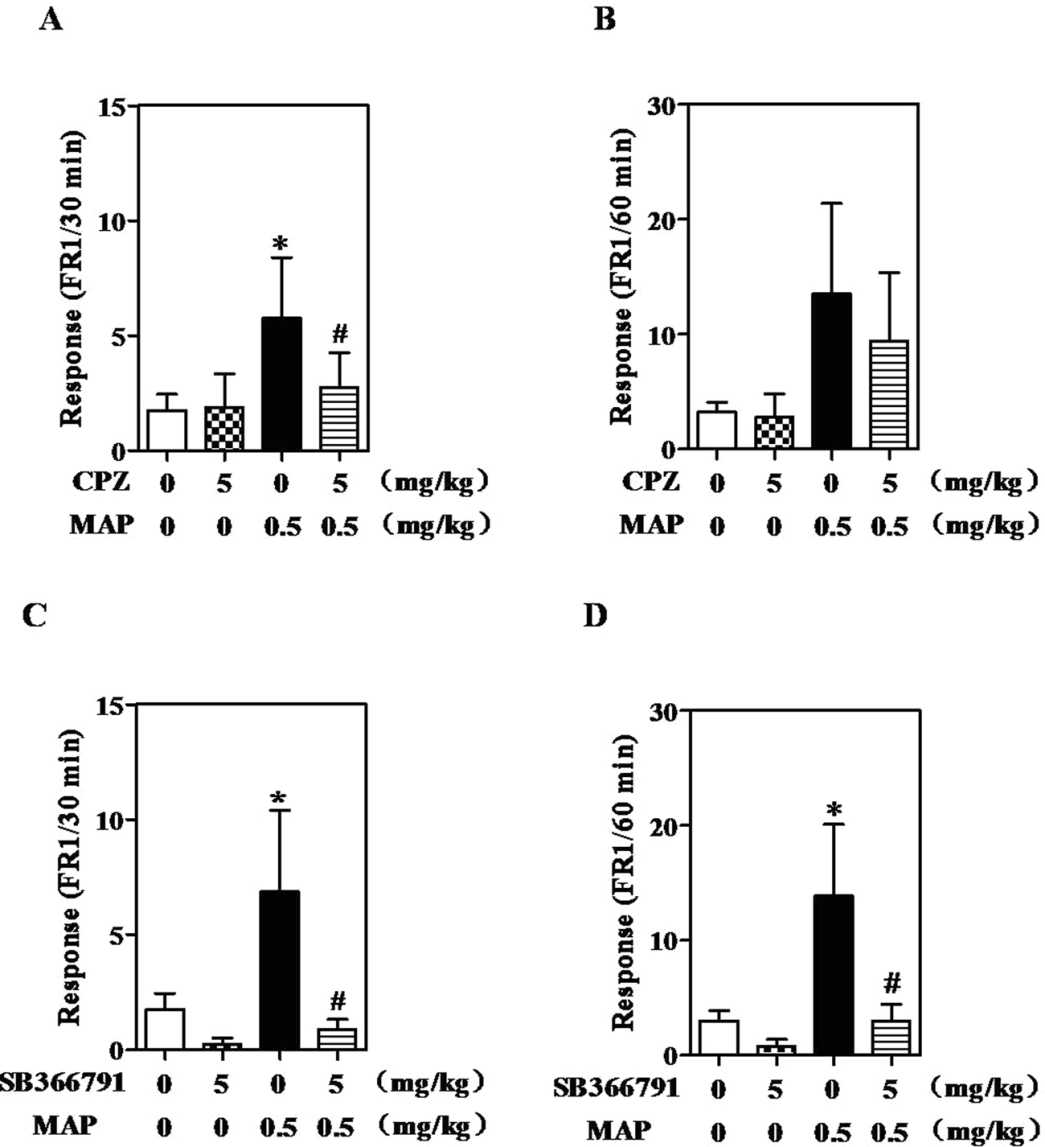
Effects of CPZ (**A** and **B**) and SB (**C** and **D**) on relapse to MAP seeking. CPZ or SB was intraperitoneally injected into rats 30 min before the reinstatement test session. Data are expressed as the number of self-administration ± SEM (n = 8). Data from the first 30 min of a 1-hr session in ShA rats under FR1 schedule are shown. Statistical analysis was performed by a repeated measurement one-way ANOVA followed by Bonferroni *post hoc* test. ^*^P < 0.05 vs. saline control group; ^#^*P* < 0.05 vs. MAP control group. MAP, methamphetamine; CPZ, capsazepine.

Pretreatment with 5 mg/kg CPZ or SB significantly attenuated MAP-seeking behavior, expressed by self-administration of MAP (right lever pressing) in the first 30 min of the MAP-primed reinstatement session (52% and 87%) under a FR 1) schedule (CPZ: F_(3,31)_ = 3.735, *P* < 0.05, Fig. 3A; SB: F_(3,31)_ = 3.210, *P* < 0.05, Fig. 3C). Furthermore, during 60 min of the MAP-primed reinstatement session, although the two antagonists demonstrated similar trends in results for the first 30 min, only SB reached statistical significance (CPZ: F_(3,31)_ = 2.251, *P = *0.1121, Fig. 3B; SB: F_(3,31)_ = 4.538, *P* < 0.05, Fig. 3D). This indicated that both CPZ and SB could suppress MAP reinstatement. In contrast, CPZ or SB alone did not affect MAP-seeking behavior in rats throughout a session under a FR 1 schedule.

### TRPV1 expressions in the NAc and DSt regions of mice exhibiting MAP-induced CPP

**Figure 4:**
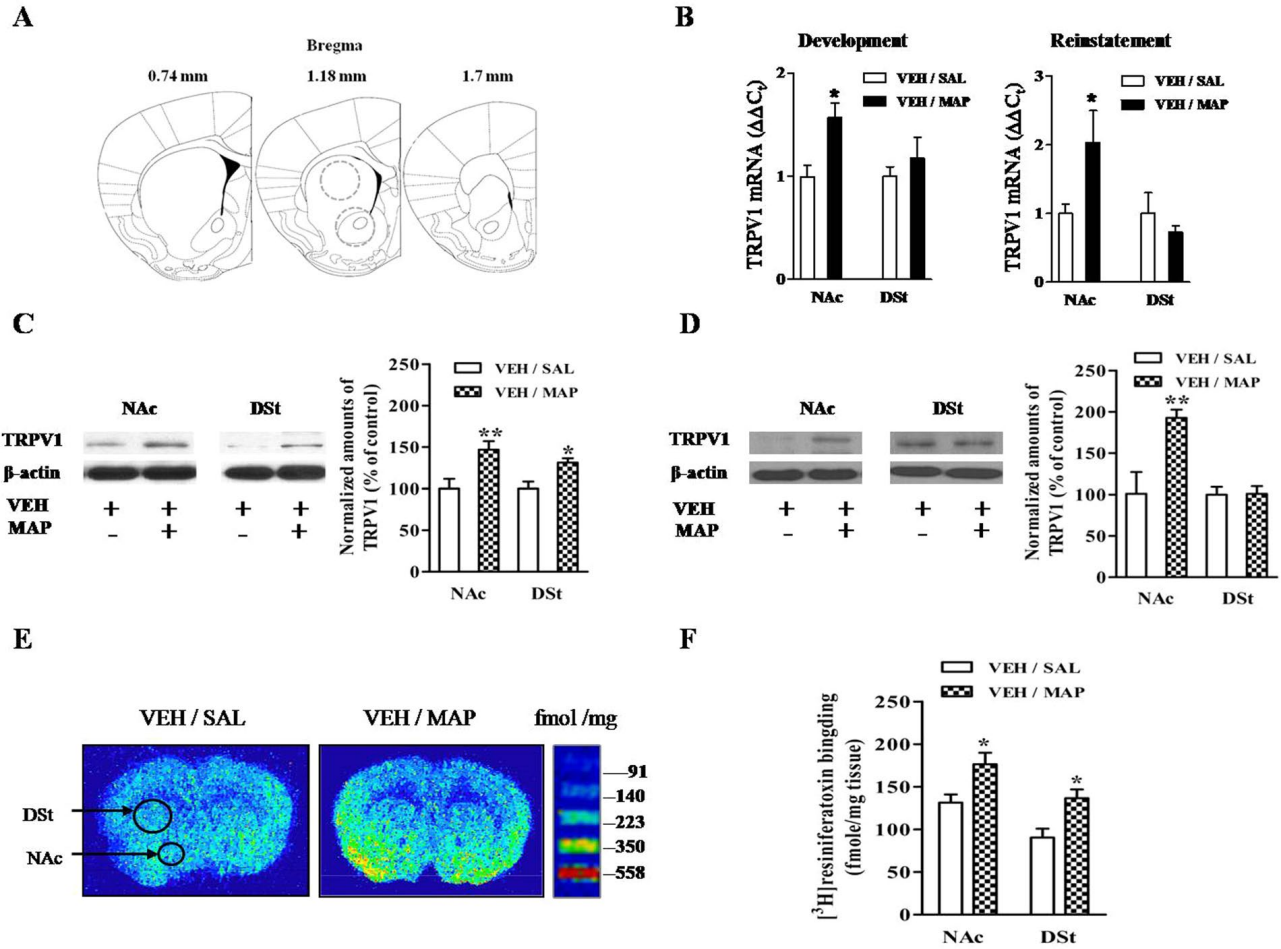
(**A**) The locations of punched samples for the NAc and DSt regions. (**B**) The expression of TRPV1 mRNA in the NAc and DSt regions after CPP development and reinstatement. (**C**,**D**) Increased TRPV1 protein expression after CPP development (n = 3–5) and drug-primed CPP reinstatement (n = 3). Full-length blots are presented in Supplementary Figure S1. (**E**,**F**) Representative autoradiograph and quantitative analysis of [^3^H] resiniferatoxin binding in the NAc and DSt regions of mice after CPP development (n = 12). Results are presented as the mean ± SEM. Statistical analysis was performed by a two-way ANOVA followed by Bonferroni *post hoc* test. ^*^P < 0.05, ^**^P < 0.01 vs. saline control group. NAc, nucleus accumbens; DSt, dorsal striatum; SAL, saline; VEH, vehicle; MAP, methamphetamine; CPZ, capsazepine.

As an initial step in assessing the relationship between TRPV1 and MAP-induced drug dependence, we examined whether repeated MAP treatment altered the abundance of TRPV1 mRNA in the NAc (n = 8–12) and DSt (n = 8–12) regions using real-time RT-PCR. We found that levels of TRPV1 mRNA were significantly higher in NAc region (*P* < 0.05), but not in the DSt region (*P* > 0.05), after both CPP development [drug, *F*_(1,35)_ = 6.456, *P* > 0.05; region, *F*_(1,35)_ = 1.731, *P* < 0.05; drug × region interactions, *F*_(1,35)_ = 1.827, *P* > 0.05; two-way ANOVA] and the reinstatement test [drug, *F*_(1,35)_ = 1.514, *P* > 0.05; region, *F*_(1,35)_ = 4.404, *P* < 0.05; drug × region interactions, *F*_(1,35)_ = 4.393, *P* < 0.05; two-way ANOVA] (Fig. 4B). These observations suggest that MAP-induced CPP was correlated to a region-specific change in TRPV1 mRNA.

To examine whether the TRPV1 protein expression was related to MAP-induced CPP, we conducted a Western blot for the NAc (n = 3–5) and DSt (n = 3–5) regions. The level of TRPV1 protein expression was significantly higher in both NAc (*P* < 0.01) and DSt (*P* < 0.05) regions of MAP-induced CPP development mice compared with the control group [drug, *F*_(1,16)_ = 18.87, *P* < 0.05; region, *F*_(1,16)_ = 0.7606, *P* > 0.05; drug × region interactions, *F*_(1,16)_ = 0.7638, *P* > 0.05; two-way ANOVA; Fig. 4C]. However, in the MAP-induced CPP reinstatement mice, the level of TRPV1 protein was up-regulated only in the NAc region (*P* < 0.01), not in the DSt region (*P* > 0.05), suggesting that up-regulation of TRPV1 in the NAc region might be involved in MAP-induced CPP only in both the development and reinstatement phases, while in the DSt region it was involved in CPP only during development [drug, *F*_(1,8)_ = 9.133, *P* < 0.05; region, *F*_(1,8)_ = 9.199, *P* < 0.05; drug × region interactions, *F*_(1,8)_ = 8.756, *P* < 0.05; two-way ANOVA; Fig. 4D].

Moreover, we also conducted a [^3^H]resiniferatoxin binding test for the NAc (n = 12) and DSt (n = 12) samples after the CPP development test. A two-way ANOVA showed significant effects for both drug [*F*_(1,36)_ = 14.63, *P* < 0.001] and region [*F*_(1,36)_ = 11.47, *P* < 0.01], with no significant drug × region interaction [*F*_(1,36)_ = 0.002, *P* > 0.05]. *Post hoc* testing showed that [^3^H]resiniferatoxin binding in the NAc (*P* < 0.05) and DSt (*P* < 0.05) regions of mice treated with MAP was significantly higher than in the control group (Fig. 4E and F).

### Effects of CPZ on DAT in the NAc and DSt regions of mice exhibiting MAP-induced CPP

**Figure 5:**
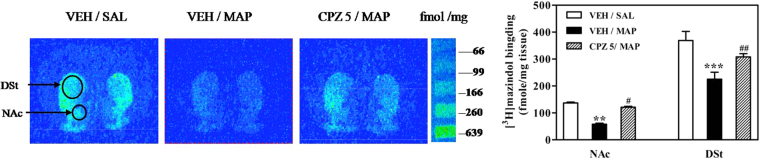
Representative autoradiograph and quantitative analysis of [3H]mazindol binding in the NAc and DSt regions of mice after CPP development. Results are presented as mean ± SEM (n = 6). Statistical analysis was performed by a two-way ANOVA followed by Bonferroni *post hoc* test. ^**^P < 0.01, ^***^P < 0.01 vs. saline control group; ^#^*P* < 0.05, ^##^*P* < 0.01 vs. MAP control group. NAc, nucleus accumbens; DSt, dorsal striatum; SAL, saline; VEH, vehicle; MAP, methamphetamine; CPZ, capsazepine.

We measured [^3^H]mazindol binding levels as readout of surface DAT activity in the NAc and DSt regions of mice after repeated MAP treatment. We found that the binding of DAT in both NAc (*P* < 0.01) and DSt (*P* < 0.001) regions of MAP-treated mice was significantly lower than saline controls. This down-regulation of DAT in the NAc (*P* < 0.05) and DSt regions (*P* < 0.01) was reversed by pretreatment with CPZ, suggesting that DAT is involved in the attenuating effect of CPZ on MAP-induced CPP development [drug, *F*_(2,54)_ = 19.08, *P* < 0.001; region, *F*_(1,54)_ = 171.7, *P* < 0.001; drug × region interactions, *F*_(2,54)_ = 1.645, *P* > 0.05; two-way ANOVA; Fig. 5].

### Effect of CPZ on the DA release induced by MAP in the NAc region

**Figure 6:**
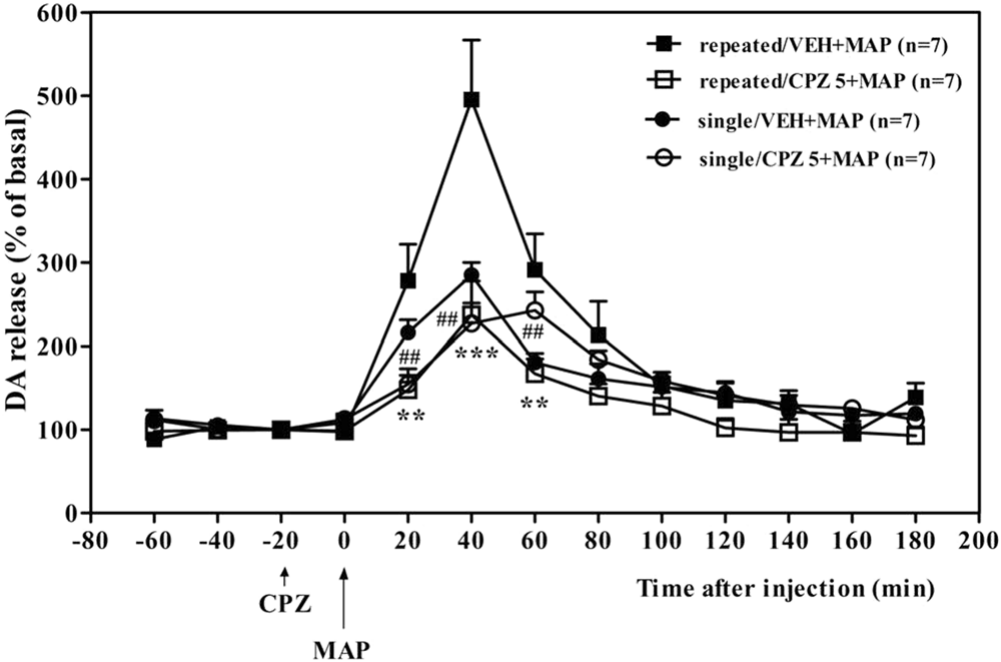
Effects of single and repeated administration of CPZ on the MAP-enhanced release of DA from the NAc region of freely-moving mice. Mice were treated with CPZ (5 mg/kg, i.p.) once every other day for seven days (days 1, 3, 5, and 7; four injections) prior to 20 min administration of either saline or MAP after the collection of three samples. Dialysate samples were collected for 20 min. The release of DA is expressed as the percent change over the mean of the first three samples, taken to be controls (mean ± SEM of at least seven different mice in each group are presented). Statistical analysis was performed by two-way repeated measures ANOVA, with the drug as an independent factor and time as a repeated factor, followed by Bonferroni’s *post hoc* test. ^**^P < 0.01, ^***^P < 0.001 vs. the repeated MAP-treated group; ^##^*P* < 0.01 vs. with the single MAP-treated group. SAL, saline; VEH, vehicle; MAP, methamphetamine; CPZ: capsazepine.

The effects of CPZ on increases in extracellular DA levels induced by single or repeated MAP treatment were examined in the NAc region of mice via an *in vivo* microdialysis technique (Fig. 6). Single and repeated MAP treatment caused a marked increase in extracellular DA levels in the NAc region compared with the saline control group (data of the saline control group are not shown). Pretreatment with CPZ (5 mg/kg, i.p.) prior to a single MAP treatment significantly decreased the MAP-induced increase in extracellular DA levels at 20, 40, and 60 min after MAP injection (*P* < 0.01). This was confirmed by a two-way ANOVA with significant primary effects of time (*F*_(12,148)_ = 31, *P* < 0.001) and absence of treatment (*F*_(1,148)_ = 0.16, *P* > 0.05) and a significant interaction between the two (*F*_(12,148)_ = 3.4, *P* < 0.001). In addition, pretreatment with CPZ prior to repeated MAP administration (on days 1, 3, 5, and 7, for a total of four injections) also dramatically attenuated the repeated MAP-induced increase in extracellular DA levels at 20, 40, and 60 min after MAP injection (*P* < 0.01). This finding was confirmed by two-way ANOVA with significant main effects of treatment, *F*_(1,148)_ = 30, *P* < 0.001, and time, *F*_(12,148)_ = 20, *P* < 0.001, and there was a significant interaction between the two, *F*_(12,148)_ = 4.6, *P* < 0.001.

## Discussion

In the present study, we performed CPP and self-administration tests to examine the effects of CPZ and SB, two competitive TRPV1 antagonists, on MAP reward. We observed that blocking TRPV1 could disrupt the MAP-induced CPP in both the induction and reinstatement phases. It has demonstrated that SB is a more selective and *in vivo* also more potent TRPV1 receptor antagonist than CPZ, based on its lower IC_50_ values in capsaicin-induced Ca^2+^ influx in cultured trigeminal ganglion cells and anti-hypothermic effects in rat^18^. These results reasonably explained why inhibitory effect of CPZ and SB on MAP-induced CPP showed different manner in our CPP data. In addition, there are varies between Fig. 1C and D on the magnitude of CPP, we suppose that it might because of experimental animal individual differences. On the other hand, to identify adverse effects of TRPV1 antagonism on non reward systems such as locomotor activity, learning and memory recall, we also conducted relevant preliminary tests. The locomotor activity data showed that three doses of CPZ (2.5, 5 and 10 mg/kg) had no effect on distance travelled, suggesting CPZ is not likely involved in impairment of motor activity. In addition, we have performed Morris Water Maze with TRPV1 KO mice and it had no change on any parameter. Consistent with our results, one recent study has demonstrated that CPZ (1 mg/kg) did not change cognitive function in Aβ42-treated mice through Morris Water Maze test^19^. Taken together, we suppose that TRPV1 antagonism had no adverse effects on motor activity and learning and memory.

To provide further evidence for the hypothesis that TRPV1 plays an important role in MAP-induced CPP, we conducted a CPP test using TRPV1 KO mice. As expected, in both males and females, mice lacking TRPV1 did not show MAP-induced CPP compared with wild type mice. These results are in line with our recent studies, which found that blocking TRPV1 could attenuate morphine-induced CPP^11,12,20^. In addition, functional studies have revealed that deletion of the TRPV1 gene in mice can affect ethanol consumption^8^, as well as anxiety and conditioned fear^7^. Moreover, blockade of TRPV1 by SB decreased cocaine-induced reinstatement of cocaine-seeking behavior^13^. It is noticeable that TRPV1 male KO mice displayed slightly decrease CPP score compared to their control group. A likely explanation for this interesting data is that TRPV1 null mice itself have increased locomotor responding to MAP and the hyperlocomotor activity could induce increase in frequency of movement of mice in CPP boxes. Similarly, one previous study has found that TRPV1 KO mice exhibit augmented cocaine-induced locomotor activity^21^. Furthermore, our preliminary locomotor activity test with TRPV1 KO mice already revealed a hyperlocomotor activity after single (day 1) or repeated treatment (day 7) of MAP compared with that of control group. Therefore, these data indicate that MAP-induced hyperlocomotor phenotype in the TRPV1 KO mice may involve in slightly reduced CPP score of TRPV1 KO mice.

It is well-known that drug self-administration procedures allow rats to regulate drug intake by eliciting a behavioral output such as a lever press or nose poke into a hole. This benefit increases face and predictive validity of the self-administration model relative to systemic drug administration. Additionally, the predictive validity of self-administration models of psychostimulant abuse liability is robust^22^. In the present study, we demonstrated that both CPZ and SB significantly attenuated MAP-seeking behavior using a MAP self-administration paradigm. The observation that TRPV1 is involved in MAP self-administration is a novel finding. Our results on the TRPV1 antagonist SB in MAP-induced reinstatement are consistent with descriptions in a previous study^13^ demonstrating that TRPV1 receptors are engaged in cocaine-seeking behavior. Together with the CPP results, this strongly supports our hypothesis that TRPV1 antagonism is involved in MAP reward.

The NAc region is known to be a critical region in MAP reward and addiction^16,23^. Previous studies have suggested that activation of TRPV1 triggers long-term depression in the NAc region^21^, and blockade of TRPV1 in the NAc region inhibited morphine CPP in rats^11^. As a component of the nigrostriatal dopaminergic pathway, the DSt region was also reported to be involved in reward-based learning^24,25^ and drug seeking behavior^12,20,23,26^. Importantly, several studies using Western blot analysis^12,18^ and the patch-clamp technique^21,27,28^ identified that the NAc and DSt regions express TRPV1. Therefore, our quantitative real-time RT-PCR, Western blot, and [^3^H]resiniferatoxin autoradiographic analyses focused on TRPV1 in the NAc and DSt regions. The results revealed that TRPV1 mRNA, protein expression and receptor binding were up-regulated in the NAc and/or DSt regions in MAP-induced CPP mice compared to a saline control group. During development of CPP, there was a significant increase in TRPV1 protein in the DSt region, but only a trend for a slight increase in TRPV1 mRNA was seen. It seems likely that TRPV1 protein is expressed on presynaptic DAT positive terminals where regulate DAT function on the basis of our data from DAT binding assay (Fig. 5). One previous study detected TRPV1 in synaptic transport vesicles and demonstrated that recycling and/or fusion of these vesicles can be rapidly modulated by TRPV1 activation, which suggests that TRPV1, as a synaptic protein, is involved in the release of synaptic transmitters^29^. Moreover, in the same paper, it was demonstrated that TRPV1 could result in exocytosis by facilitating Ca^2+^ influx^29^. From this, we hypothesized that activation of TRPV1 in the NAc or DSt region in MAP-induced CPP mice promoted DA vesicle fusion by inducing direct Ca^2+^ influx and led to DA release at synapses. Therefore, the increase in DA caused by activation of TRPV1 in the NAc or DSt regions was positively correlated with MAP reward. However, the exact mechanisms involved in TRPV1 antagonism on MAP reward are not fully understood.

In our opinion, at least two possible mechanisms are involved in TRPV1 antagonism on MAP-induced CPP. First, inhibition of TRPV1 can diminish the release of DA by MAP. In the present microdialysis study, both single and repeated pretreatment with 5 mg/kg CPZ significantly reduced the MAP-enhanced release of DA in the NAc region. These results also strongly support our hypothesis that TRPV1 contributes to enhancement of DA release by MAP at synapses. Therefore, inhibition of TRPV1 in the NAc region might modulate synaptic dopaminergic functions and significantly decrease DA release. Besides DA, we have considered that anandamide, as an endogenous activator of TRPV1, could be involved in MAP addiction. As expected, previous studies already demonstrated that the anandamide level decreased in the NAc region after amphetamine-induced behavioral sensitization^30^ and repeated MAP administration^31^. On the contrary, it increased in the DSt region after repeated amphetamine treatment^30^. Based on these results, to completely delineate the role of TRPV1 antagonism in MAP addiction, our future study will focus on repeated MAP treatments to test whether there is a change in the anandamide level in the NAc or DSt region. Second, the inhibition of TRPV1 can restore abnormal DAT function caused by repeated MAP treatments and contribute to the stability of DA levels at synapses. In the present study, we found that repeated administration of MAP reduced DAT binding densities in the NAc and DSt regions in MAP-treated mice, and pretreatment with CPZ significantly reversed these decreases, indicating that the attenuating effects of CPZ in MAP reward were mediated by DAT in the DA system. It is well-known that MAP is an indirect agonist of the catecholaminergic systems. MAP releases catecholamines from nerve terminals, like DA, and this process involves a reversal of the DAT^32^. One previous study showed that four injections of MAP (0.5–2.0 mg/kg) could cause damage to DA terminals and a reduction in DAT density in nonhuman primates^33^. Furthermore, another study supported a key role for TRPV1 in hyperdopaminergic-related hyperactivity. This study found markedly decreased anandamide levels in the striatum of DAT KO mice; they then used three different indirect endocannabinoid agonists to show that these could attenuate spontaneous hyperlocomotion in DAT KO mice, and that this hyperlocomotion was significantly attenuated by the TRPV1 antagonist CPZ. It is very important to note that TRPV1 receptor binding by [^3^H]resiniferatoxin was greater in the striatum of DAT KO mice^34^. This finding is consistent with our results that attenuated DAT binding by MAP was recovered by pretreatment with CPZ. Collectively, these findings indicated a correlation between TRPV1 and DAT in MAP reward, as DAT KO mice were hyperdopaminergic, hyperactive, and display perturbed cognitive performance^34^, and these properties were very similar to MAP-induced behavioral changes. Conversely, to assess the original DAT level of TRPV1 KO mice brain, we have conducted preliminary [^3^H]mazindol binding assay with TRPV1 null mice, however, male TRPV1 KO mice did not show any change in NAc and DSt regions (data are not shown). Therefore, the precise mechanisms for this process need to be further elucidated in future studies.

The present study is the first to show that TRPV1 antagonists effectively blocked CPP and self-administration induced by MAP and demonstrated that MAP-induced TRPV1 up-regulation in the NAc and DSt regions significantly contributed to MAP reward and reinstatement. Furthermore, we found that increased DA release and attenuated DAT function accompanied MAP reward via TRPV1. These results indicate that TRPV1 plays a key role in MAP reward and reinstatement, and thus represents a promising novel pharmacological target for treating MAP addiction.

## Methods

### Animals

CD1 male mice (20–22 g, 4 weeks old) were purchased from Dae Han Biolink Co., Ltd (Eumseong, Republic of Korea). Male and female C57BL/6J mice and TRPV1 KO mice were obtained from Jackson Laboratories (Bay Harbor, ME, USA). The C57BL/6 J mice and KO mice (20–25 g, 9–16 weeks old) were used after eight generations. Thirty-one male Wistar rats (250–275 g; Charles River, Hollister, CA, USA) were used for self-administration. They were acclimatized under controlled temperature (22 ± 2 °C) and light (12–12 h light/dark cycles) with free access to food and water. All procedures involving the use of animals were performed in strict accordance with the U.S. National Institutes of Health (NIH) Guide for the Care and Use of Laboratory Animals, and the study protocol was approved by the Animal Care and Use Committee of Sungkyunkwan University in Suwon, Republic of Korea.

### Reagents

Methamphetamine hydrochloride was purchased from the United States Pharmacopeial Convention (USP Inc., Rockville, MD, USA). Capsazepine (CPZ; 2-[2-(4-chlorphenyl) ethylamino-thiocarbonyl]-7,8-dihydroxy-2,3,4,5 tetrahydro-lH-2-benzaze-pine) and SB366791 (SB; *N*-[3-methoxyphenyl]-4-chlorocinnamide) were purchased from Tocris (Tocris Cookson Ltd., Bristol, UK). MAP was dissolved in physiological saline. CPZ and SB were dissolved in saline containing 10% dimethyl sulfoxide (DMSO) and 10% Tween 80 (Sigma Aldrich, St. Louis, MO, USA). All drugs were freshly prepared and administered intraperitoneally (i.p.) at 10 mL/kg. The radiolabeled compounds [^3^H]resiniferatoxin (37.2 Ci/mmol) and [^3^H]mazindol (19.8 Ci/mmol) were purchased from Perkin-Elmer (Boston, MA, USA).

### CPP procedure

#### Development of MAP-induced CPP

The CPP apparatus consisted of two square-based Plexiglas compartments (15 × 15 × 15 cm), one with white walls and the other with black walls, which could be isolated by guillotine doors. To provide a tactile difference between the compartments, the black compartments had black meshed floors, while the white compartments had white smooth floors. A computer-based video-tracking system (NeuroVision, Pusan National University, Pusan, Korea) was used to record and analyze the behavioral data. The time spent in each compartment of the CPP apparatus was tracked automatically^35^. The CPP paradigm was performed using a previously established procedure with a minor modification^35–38^. The CPP test consisted of four phases: habituation, pre-test, conditioning, and post-test. During the first phase, animals were habituated to the apparatus by being placed in a compartment designated as the start compartment and allowed to freely explore both chambers for 60 min each day for 4 days. The start compartment was counterbalanced, with half of the mice starting on the left side of the conditioning compartment and the other half starting on the right side. On the 5^th^ day, baseline preferences (pre-test) were assessed by placing the animals in a tunnel in the central part of the CPP apparatus with both guillotine doors open, allowing the animal to freely access both compartments for 15 min. Time spent in each compartment was recorded. Pre-test data were analyzed and showed no significant differences between the groups. The conditioning phase occurred over a period of 8 days. During this phase, the guillotine doors were closed. Within each group, the drug-paired compartment was counterbalanced, such that half of the mice were confined to the black compartment, while the other half were confined to the white compartment. On days 6, 8, 10, and 12, the mice received MAP (1 mg/kg) or saline 30 min after administration of CPZ 2.5 or 5 mg/kg, SB 0.3 or 0.6 mg, or vehicle, and then the animals were confined in the one compartment for 60 min. On days 7, 9, 11, and 13, the mice received saline just prior to being confined in the other compartment for 60 min. During the post-test phase, the guillotine doors were opened, and the untreated mice were placed in the tunnel in the central part of the apparatus. The time that the mice spent in each compartment of the CPP apparatus was recorded for 15 min. CPP scores were expressed as the difference between the baseline test and post-test times during which the mice were in the drug-paired compartment.

#### Extinction of MAP-induced CPP

A different set of mice was conditioned for development of MAP-induced CPP. During the extinction phase, the mice were administered saline and immediately placed in the tunnel in the central part of the CPP apparatus with both guillotine doors open, allowing the animal to freely access both compartments for 60 min. The criterion to consider the preference to be extinguished was the lack of statistical significance between the time spent by the animals of each group in the drug-paired compartment and that of the pre-test session.

#### Drug-primed reinstatement of MAP-induced CPP

After the extinction phase, the mice were injected with CPZ or SB 30 min prior to a priming injection of MAP and were allowed to move freely between the two compartments. The time each mouse spent in each compartment was recorded for 15 min. CPP scores were expressed as the difference in the pre-test and drug-primed reinstatement test times during which the mice were in the drug-paired compartment.

### Self-administration

#### Apparatus

During experimental sessions, each rat was placed in an operant chamber, which was placed in a light and sound-attenuating cubicle (28 × 26 × 20 cm; Med Associates Inc., St Albans, VT, USA). Each chamber was equipped with response levers (4.8 × 1.9 cm), a cue light (3 W, 28 V), and a house light (3 W, 28 V). A cue light was located above each response lever. The front door and the back wall of the chamber were made of transparent plastic, and the other walls were opaque metal. Drug injections were delivered by a syringe pump (Razel Scientific Instruments, Georgia, VT, USA) located on top of the cubicle. Experimental sessions were controlled and recorded by a PC computer with a custom interface and software in the experimental room. At the start of each session, two response levers were placed in the chamber, and one response on the active lever resulted in the delivery of 0.1 ml of a drug solution over 4 s (FR1 schedule). The cue light above the active lever was illuminated at the onset of each infusion and remained illuminated throughout the time-out period (20 s), during which responses were recorded without consequence. The darkening of the cue light signaled the availability of the next infusion.

#### Food pellet training

To facilitate the acquisition of operant responding, rats were initially trained to press a lever to receive 45 mg of food pellets (Bio-Serv, Frenchtown, NJ, USA) until criteria were satisfied (80 food pellets for three consecutive days) in 1-h daily sessions.

#### Surgery

Rats were anesthetized with 2–3% of isoflurane in oxygen. They were implanted with a silastic catheter (0.3 × 0.64 mm OD; Dow Corning Co., Midland, MI, USA) into the right jugular vein under aseptic conditions. The distal end of the catheter was threaded to the back of the rat, where it exited the body via a metal guide cannula (22 G, Plastics One Inc., Roanoke, VA, USA) that was anchored at the back of the rat. Immediately, after surgery, rats were given analgesics (Flunixin®, 2.5 mg/kg, s.c.). The rats were subjected to antibiotic (Timentins, 20 mg, i.v.; SmithKline Beecham, Philadelphia, PA, USA) for seven days after the surgeries. The catheter was flushed daily with heparinized saline (30 U/ml). The patency of the rat catheters was tested using methohexital sodium (Brevital®, 10 mg/ml, 2 mg/rat) whenever a catheter failure was suspected during the study. Generally, a total loss of muscle tone within 3 s after a methohexital injection indicated the patency of a catheter.

#### Acquisition of MAP self-administration

Following the recovery period from surgery, rats were trained on a FR1 schedule of MAP self-administration (0.05 mg/kg/infusion) in daily 2-h sessions for 8–10 days. When rats showed a stable response (defined as average values of three consecutive sessions having less than 20% variation), the effects of SB on MAP self-administration were tested on a FR1 schedule for two consecutive days. Half an hour before testing, each rat received SB (5 or 10 mg/kg) or vehicle in a volume of 1 ml/kg.

#### Relapse of MAP self-administration

After surgery and recovery, the rats were trained to self-administer 0.05 mg/kg/infusion MAP in 1-h sessions under a FR 1 schedule for 20 sessions. Sessions were performed 6–7 days per week. All rats maintained catheter patency throughout the course of the self-administration experiment.

Following MAP self-administration and before the first reinstatement test, rats underwent daily 1-h extinction sessions. During each session, responses on both levers were recorded but had no consequences. Once active lever pressing was extinguished to a minimum of seven extinction sessions with ≤25 active lever responses per session for two consecutive days (data not shown), animals underwent MAP-primed (0.5 mg/kg, i.p.) reinstatement tests. Thirty minutes before each reinstatement test, each rat received CPZ or SB (5 mg/kg, i.p.) or vehicle. During cue-induced reinstatement testing, active lever presses resulted in the presentation of light + tone CS in the absence of MAP reinforcement.

### Tissue preparation

After the MAP CPP test, mice were sacrificed by decapitation. Mice brains were quickly removed and frozen in dry ice. A coronal section (bregma +1.7 to +0.74 mm) was cut according to the mouse brain atlas (Fig. 5A). The Nucleus accumbens (NAc) and dorsal striatum (DSt) regions were punched bilaterally using a Harris Uni-Core 1.20 (TedPella, Inc., Redding, CA, USA). These brain tissues were used for quantitative real-time RT-PCR and western blot assays.

### Quantitative real-time RT-PCR

Total RNA was isolated from tissues using the RNeasy Micro kit (Qiagen, Valencia, CA, USA) and reverse-transcribed with the SuperScript™ III First-Strand Synthesis System for RT-PCR (Invitrogen, Carlsbad, CA, USA). Quantification of mouse TRPV1 was performed with a Rotor-Gene 6000 real-time amplification system (Corbett Research, Mortlake, NSW, Australia). PCR was conducted with a SensiMixPlus SYBR Kit (Quantace, Alexandria, NSW, Australia). Relative quantification started from 300 ng of RNA with a single-enzyme RT-PCR in a total reaction volume of 20 μl. The RT-PCR cycling conditions involved holding at 95 °C for 10 min, followed by 45 cycles of PCR amplification with denaturation (95 °C for 10 s), primer annealing (58 °C for 15 s), and extension (72 °C for 20 s) with TRPV1 primers [5′-AGCCATGCTCAATCTGCAC-3′ as the forward primer (FP) and 5′-TGCTGTCTGGCCCTTGTAG-3′ as the reverse primer (RP)]. Amplification data were acquired in the extension step and analyzed with Corbett Research Software version 1.7.75 using the comparative critical threshold (ΔΔCt) values. Target gene levels were normalized to that of β-actin (5′-AGAGGGAAATCGTGCGTGAC-3′ as FP and 5′-CAATAGTGATGAC-CTGGCCGT-3′ as RP).

### Western blot

The NAc and DSt regions from three mice were punched and pooled after behavioral experiments; homogenized in 10 mM Tris buffer (pH 7.5) containing 160 mM sucrose, 1 mM EDTA, 1 mM DTT, a protease inhibitor cocktail tablet (Complete Mini, Roche Diagnostics, Indianapolis, IN, USA), and a phosphotase inhibitor cocktail tablet (PhosSTOP, Roche Diagnostics, Indianapolis, IN, USA); and centrifuged at 15,000 rpm for 20 min. Proteins from supernatant were used in Western blotting. Fifty micrograms of protein from tissue homogenates were separated on 6% and 12% SDS–PAGE gel, transferred onto a PVDF membrane, blocked with 5% BSA in TBS containing 0.05% Tween-20, and incubated with rabbit anti-TRPV1 (1:3000; Abcam, Cambridge, UK), followed by HRP-conjugated rabbit secondary antibody (1:5000; Cell Signaling, St Louis, MO, USA). Loading of comparable mouse brain samples was confirmed by blotting with anti-mouse β-actin antibody (Sigma Aldrich, St. Louis, MO, USA). For quantitative analysis of TRPV1, the density of TRPV1 protein detected with anti-TRPV1 antibody was divided by the density of beta actin protein detected with anti-beta actin antibody. The value was then determined after being normalized to the value of the saline control group.

### Quantitative receptor autoradiography

For all ligand-binding studies, the mice used were killed by decapitation immediately after testing. The brains were rapidly removed and dissected, frozen on dry ice for 5 min, and stored at −80 °C until processed. Coronal sections (20 μm thick) were cut using a cryostat (Leica, Wetzlar, Germany). Equivalent sections for all brains were collected at three different levels, which allowed the authors to map different brain areas along the rostrocaudal axis. The section level was 0.8–0.9 mm (Nucleus accumbens, NAc; dorsal striatum, DSt) from bregma according to the Paxinos atlas^39^. Sections were immediately mounted on 1.5% gelatin-coated slides, dried for 30 min at room temperature, and stored at −80 °C until use.

In order to investigate [^3^H]resiniferatoxin binding for TRPV1^40^, the sections were allowed to equilibrate at room temperature and then incubated in an assay buffer consisting of 10 mM HEPES, 5 mM KCl, 5.8 mM NaCl, 0.75 mM MgCl_2_, 320 mM sucrose, and 1 mg/ml BSA (pH 7.4) containing 1 nM [^3^H]resiniferatoxin for 60 min at 37 °C. Nonspecific binding was defined by the addition of 1 μM unlabeled resiniferatoxin to a parallel series of sections. Following incubation, sections were washed four times in 20 mM Tris–HCl (+0.1% BSA) for 10 min at 4 °C, rinsed in 1 mg/ml α-acid glycoprotein (orosomucoid; Sigma Aldrich, St. Louis, MO, USA) at 4 °C, and dipped into distilled water at 4 °C to remove buffer salts.

Autoradiograms were generated as described previously for DAT binding with a slight modification^41,42^. In brief, for DAT binding, the sections were preincubated for 10 min at 4 °C in 50 mM Tris-HCl buffer (pH 7.4) containing 30 mM NaCl and 5 mM KCl and were then incubated for 1 h at 4 °C in the same preincubation buffer in the presence of 4 nM [^3^H]mazindol. Nonspecific binding was assessed in the presence of 1 mM mazindol for DAT. After incubation, all slide series were rinsed twice in 50 mM Tris-HCl buffer for 5 min at 4 °C, followed by one brief wash in an excess of cold distilled water.

After washing, all slides were gently air-dried and stored overnight in a desiccator at room temperature. Kodak BioMax MR film was developed after being exposed to [^3^H]resiniferatoxin for 5 weeks and [^3^H]mazindol for three weeks in cassettes, together with a set of tritium standards ([^3^H]Microscale™, Amersham) at 4 °C. Autoradiograms were analyzed by a digital scanner using the image acquisition and analysis program Image Quant 3.3 (Molecular Dynamics, Sunnyvale, CA, USA). Standard curves from standard [^3^H]Microscales™ were used to convert density levels to fmol per milligram of wet brain tissue. The mean density was measured from at least two sections per region in each mouse.

### *In vivo* microdialysis

For analysis of the release of DA, animals were anesthetized with sodium pentobarbital (50 mg/kg, i.p.), and a guide cannula (CMA/7, Cupr CMA Microdialysis AB, Stockholm, Sweden) was implanted in the NAc (+1.5 mm anteroposterior, +0.8 mm mediolateral to the bregma, −4.0 mm dorsoventral to the surface of the skull)^43^. The mice were used in the microdialysis perfusion experiments three days after cannula implantation in order to give them time to recover from the surgery and allow the anesthesia to clear their systems. The probe (CMA/7 7/1, 1 mm, Cupr CMA Microdialysis AB, Stockholm, Sweden) was inserted through the guide cannula under ether anesthetic and was perfused with artificial cerebrospinal fluid (aCSF; 150 mM NaCl, 3 mM KCl, 1.4 mM CaCl_2_, 0.8 mM MgCl_2_, pH = 7.4) at a flow rate of 0.8 μl/min. During the experiments, the perfusate samples, which were collected at 20 min intervals (16 μl) in freely moving mice, were directly administered using an injection (HPLC-ECD) system for separation and quantification of DA. The mobile phase contained 75 mM NaH_2_PO4·H_2_O, 1.7 mM 1-OSA, 25 μM EDTA, 0.714 mM triethylamine, and 10% acetonitrile, pH = 3.0 and was maintained at a flow rate of 0.6 ml/min. Basal DA levels were monitored over 3 h for stabilization. Twenty minutes prior to the injection of MAP, mice underwent pretreatment [CPZ (5 mg/kg, i.p.) or saline (0.1 ml/10 g, i.p.)], and the release of DA was measured for up to 180 min. After the completion of microdialysis experiments, the mice were deeply anesthetized with pentobarbital (60 mg/kg, i.p.) and killed by decapitation. The position of probe placement was histologically verified to be in the NAc region.

### Statistical analysis

Data are expressed as the mean ± SEM. Analysis of CPP data was performed using one-way ANOVA, followed by the Newman-Keuls *post hoc* test. Self-administration data were analyzed by one-way ANOVA, followed by the uncorrected Fisher’s LSD test, repeated measures ANOVA, followed by the Bonferroni’s multiple comparison tests or the uncorrected Fisher’s LSD test. Results from quantitative real time RT-PCR, Western blotting, and quantitative autoradiography analyses were analyzed by two-way ANOVA with factors of drug and region, followed by Bonferroni *post hoc* tests. For *in vivo* microdialysis studies, values were calculated as the percent change from dialysate basal concentrations, with 100% defined as the average of three fractions prior to treatment. Microdialysis data analyses were performed using two-way ANOVA, followed by the Bonferroni’s multiple comparison tests, with treatment as the inter-subject factor and repeated measures with time as the intra-subject factor. The criterion for significance was *P* < 0.05 in all statistical analyses.

## Electronic supplementary material

Supplementary Figure S1
