# Supplementary material for: Blockade of TRPV1 Inhibits Methamphetamine-induced Rewarding Effects
Source: Sci Rep. 2018 Jan 17;8:882. doi: 10.1038/s41598-018-19207-2 (PMC5772440; doi:10.1038/s41598-018-19207-2)
Supplement: Supplementary file 1 — Supplementary Figure S1 [file 41598_2018_19207_MOESM1_ESM.pdf]

Blockade of TRPV1 Inhibits Methamphetamine-induced Rewarding Effects

Yu-Hua Tian[1,2], Shi-Xun Ma[2], Kwang-Wook Lee[2], Sunmee Wee[3], George F. Koob[3, 4], Seok-Yong Lee[2], *Choon-Gon Jang[2]

[1]Department of Pharmacology, School of Pharmacy, Qingdao University, Qingdao, 266021, China. [2]Department of Pharmacology, School of Pharmacy, Sungkyunkwan University, Suwon, 16419, Republic of Korea. [3]Committee on the Neurobiology of Addictive Disorders, The Scripps Research Institute, La Jolla, CA 92037, USA. [4]Neurobiology of Addiction Section, National Institute on Drug Abuse, National Institutes of Health, Baltimore, MD 21224, USA.

To whom correspondence should be addressed:

Choon-Gon Jang, email: jang@skku.edu

First authors: Yu-Hua Tian and Shi-Xun Ma contributed equally to this work

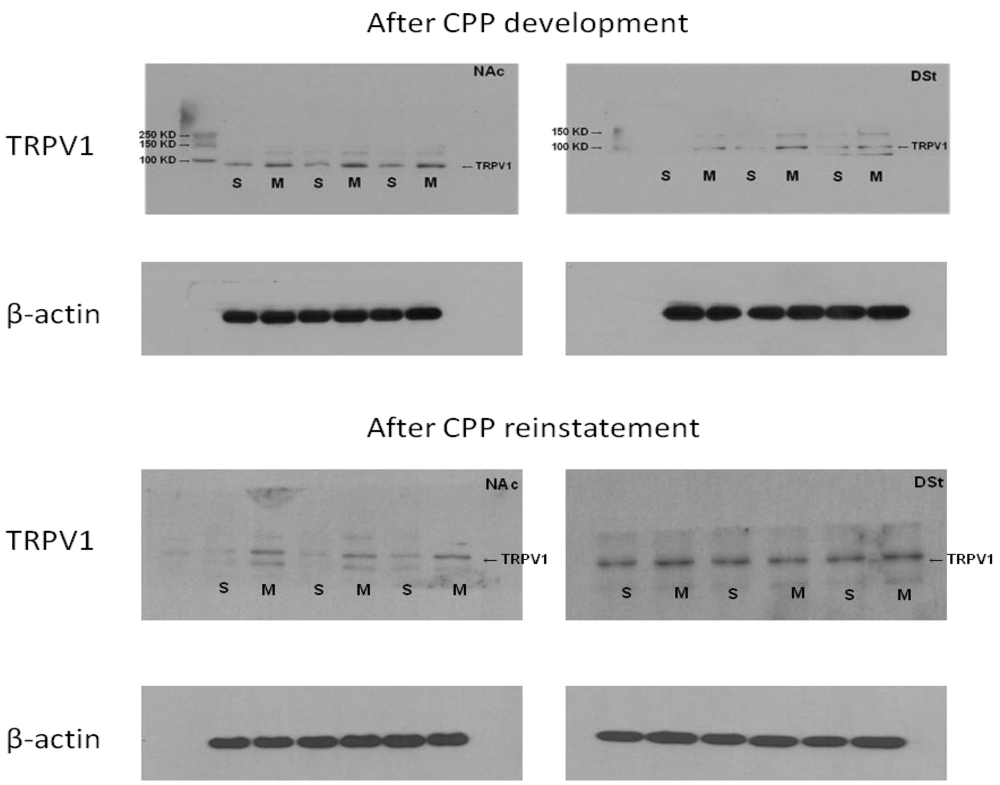

Supplementary Figure S1. Full length blots for expression of TRPV1 protein in NAc and DSt regions of mice after CPP development and reinstatement. These raw films showed that the TRPV1 protein band was located at about 95 KDa in a thin band in the film (S: saline, M: methamphetamine) (n = 3). The entire MAP group showed increased expression of TRPV1 in NAc region after both CPP development and reinstatement phase compared with saline control group. However, in DSt region, increased expression was only observed after CPP development phase.
